# Mental Hospital Magazines: The Existing Product and a Gap to Be Filled

**Published:** 1914-02-21

**Authors:** 


### MENTAL HOSPITAL MAGAZINES.

### The Existing Product and a Gap to be Filled.

It may be truly said in regard to the treatment of the insane that as the times have changed so have our ideas in regard to the therapeutics of insanity changed also; and one sign of the times is the growth of that special branch of literary venture called the mental hospital magazine. Not so many years ago anyone who should have suggested such magazines would have met with just about as much encouragement as Pinel was given when he suggested doing away with manacles and other forms of restraint. Now these magazines, if not quite as frequently met with as are libraries in mental hospitals, are yet commendably often to be found. They are part of that general amelioration in treatment which, inaugurated by Pinel and Samuel Tuke and carried on by Esquirol, Gardiner Hill, and Connolly, is, it is hoped, being brought nearer to perfection in our own time. Although Pinel does not, as far as I am aware, actually suggest the production of such magazines by the aid of the patients, he is fully cognisant of the benefit to be derived from once more endeavouring to cultivate the patient's interest in literature when convalescence has commenced, and when, as he says in his *Traité sur la manie*, "the patient's attention begins to direct itself again to the fine arts, to science, and to literature."

### Where Improvement is Needed.

The interest even of sane people must of necessity be circumscribed. Our own doings or those of our particular coterie bulk most largely in our view. Distance, perhaps because it may lend enchantment, certainly diminishes the clearness of our perceptions: consequently, there are comparatively few who by cultivation of their powers have rendered themselves capable of concentrating their attention upon distant objects. Still fewer are those who are able to dwell for any length of time among abstract ideas. The gossip of the day about our neighbours is a topic which interests the majority; and generally the doings of our own small world compel most readily our attention. Thus does the newspaper recording local doings thrive; and in this way also does an institutional magazine serve as a record of happenings which, too insignificant as they are to be noted by newspapers or journals outside the establishment, are yet a source of much interest to those within its walls. Thus we find recorded the various happenings, concerts, dances, cricket matches, and so on—trivial enough affairs so far as the great world is concerned, but *the* events from the standpoint of the inmates.

These records have, too, a certain value apart from the interest they arouse in the small circle to which their primary appeal is made. Those in authority glancing through the pages of the magazines of kindred institutions may derive information as to how to extend the range of amusements in their own hospital; how to vary the Christmas entertainments so as to obviate any possibility of monotonous repetition; hints as to fancy-dress dances; variations in the programme at the annual sports; what competitions may be instituted in order to foster most fitly friendly rivalry; what concert-parties give the greatest satisfaction; how a library appeals to the inmates; and so on over a numerous range of subjects. It may be added that this is hardly the situation of affairs at the present time. Mental hospital magazines have not yet arrived at a stage when so great a degree of utility can be claimed for them; but it is to be hoped that as time goes on there will be an extension of effort in the direction of improving the magazines which exist at present, and an inauguration of new magazines where there is a possibility of so doing.

The number of magazines and papers is already too great, it may be argued, and that may be true in so far as it applies to those which appeal to the general public. The institutional magazine is, however, in quite a different category. It does not of necessity deal with affairs generally, though these may be touched upon incidentally; and then only with those as a rule which reflect the brighter side—at least in the mental hospital magazine. There is enough of tragedy within the walls—though not the amount of it that the average lay person imagines—to make the importation of harrowing details from without a work of supererogation. In any case, it is difficult to keep the unpleasant items of news out; nay, it is impossible, as anyone will soon find out whose duties necessitate his walking frequently through the wards of a mental hospital, and who has, therefore, opportunities of conversing with the patients.

### Literary Possibilities.

It is desirable that the general tone of such magazines should be one of cheerfulness; and the endeavour should be made to exclude as far as is possible everything dealing with insanity—even the humorous side of insanity: and that insanity may be looked at from an exceedingly humorous point of view no one who has ever read "The Lunatic at Large" can deny! But even more than any effect that it might have in wounding the feelings of the patients, such a way of looking upon insanity should be tabooed because of the influence which it may have upon the nursing staff, especially the junior members, who should not in any way, however indirect, be encouraged to treat their patients as laughing-stocks. The spirit which animates anyone to laugh at the misfortunes of others is the same spirit which in former times initiated or countenanced ill-treatment.

Apart from the utilitarian aspect of such magazines and from their value as records of the doings in the establishments where they are produced, there is the consideration of them from the purely literary point of view. Reasoning *a priori* and bearing in mind the teachings of that school which would have us believe that genius has its roots in insanity, we should expect that the contributions submitted to the editor of a mental hospital magazine would be brilliant, epigrammatic, highly original, and probably of great literary value. It is another misconception—one of the many existing in regard to mental disease. It may be agreed that both genius and insanity are dependent on a specific condition—nervous instability; but that is far from saying that genius springs from insanity. Indeed, it would be more logical to say that insanity arose from genius—whatever that word may mean! —because history goes to prove that the man of genius has frequently ended in an insane condition; the "genius" being exhibited first. But that is too large a question to be entered upon here. This much may, however, be said, that experience goes to prove that the more pronounced do the signs of insanity become the less able is the individual to perform intellectual operations. The law of the dissolution of the nervous system, so ably educed by Hughlings Jackson, that the latest mental acquisitions, being the least organised, are the first to be disintegrated and to be lost, is only too lamentably true.

### The "Sanity of Art."

It may, of course, be answered that much admirable work in literature and in art has been done by those who were obviously at the time insane. This is no doubt true; but it is not argued that because a person is insane that therefore he has lost all capacity for performing his accustomed tasks. Such an argument might be good in law, but it is not valid scientifically. Mental degradation may, however, be extremely slow in certain cases; just as in others it is rapid, so rapid as to be noticeable by others than trained observers. Though slow, the process of disintegration is, however, sure; and it may be taken as axiomatic that the greater the degree of insanity the less is the intellectual power.

It is curious to observe, too, how certain attainments disappear. Literary skill is earliest impaired, especially that aspect of it which reason enters into; so that the writer who has been able to deal with his subject in a direct, forceful, and reasoned manner tends to become more vague, diffuse, and speculative. That way mysticism lies; and William Blake and Emanuel Swedenborg are examples from among men of genius of what may be seen every day in mental hospitals. The artistic power is retained longer; as this is gradually lost the more rudimentary styles replace the more cultivated one; and, whatever be the mental states of the Post-Impressionist painters, some members of that school have produced pictures which inevitably provoke contrast with the artistic productions of the insane.

### "Room for Another."

The contributions by patients to a mental hospital magazine may, therefore, vary very remarkably in their literary or artistic values. They do indeed vary within wider limits than those submitted to the editor of the ordinary magazine. Of course, the articles which do appear have had to submit to the editorial blue-pencil; whilst there are many which do not reach the printer's hands, it is almost needless to state. There is room for yet another magazine—one which should find space for the "great rejected." In it would appear the contributions—literary or artistic—which are characteristically insane. It is a scientific desideratum; and, though much of what appeared might be thought to be worthless, incoherent, and even ludicrous, it would provide interesting material for those who wish to equip themselves fully as literary critics. Some contributions of this nature are to be found in print, of course, but at present they are too scattered. The lucubrations of the monomaniac are familiar even to the newspaper reader.

Apart from any literary or artistic value, the mental hospital magazine is, however, useful as an object of interest to the patients. It is something pertaining to themselves by some of themselves, or by some who are intimately in contact with them. Prevented as many of them are from participating in the doings of the world which lies outside the walls, it is right and proper that no step should be neglected which may lead away from monotony and undue introspection. A bright and cheerful magazine may well prove its utility in this direction. H. J. N.

